# A Recombinant Alginate Lyase Algt1 with Potential in Preparing Alginate Oligosaccharides at High-Concentration Substrate

**DOI:** 10.3390/foods12214039

**Published:** 2023-11-06

**Authors:** Qingping Liang, Youtao Huang, Zhemin Liu, Mengshi Xiao, Xinmiao Ren, Tianhong Liu, Hongyan Li, Dongxing Yu, Ying Wang, Changliang Zhu

**Affiliations:** 1 College of Food Science and Engineering, Ocean University of China, Qingdao 266404, China; preenyliang@126.com (Q.L.); huangyoutao_ouc@163.com (Y.H.); 18227591863@163.com (M.X.); m13992623179@163.com (X.R.); 2 Fundamental Science R&D Center of Vazyme Biotech Co., Ltd., Nanjing 210000, China; ocean2013@126.com; 3 Marine Science Research Institute of Shandong Province, Qingdao 266003, China; oucthl@126.com (T.L.); aqlhy2008@126.com (H.L.); 4 Municipal Engineering Research Center of Aquatic Biological Quality Evaluation and Application, Qingdao 266104, China; 5 SOHAO FD-TECH Co., Ltd., Qingdao 266700, China; office@sohaofd.com

**Keywords:** alginate lyase, enzymatic activity, hydrolysis products analysis, oligosaccharides with low DP, high-concentration substrate

## Abstract

Alginate lyase has been demonstrated as an efficient tool in the preparation of functional oligosaccharides (AOS) from alginate. The high viscosity resulting from the high concentration of alginate poses a limiting factor affecting enzymatic hydrolysis, particularly in the preparation of the fragments with low degrees of polymerization (DP). Herein, a PL7 family alginate lyase Algt from *Microbulbifer thermotolerans* DSM 19189 was developed and expressed in *Pichia pastoris*. The recombinant alginate lyase Algt1 was constructed by adopting the structural domain truncation strategy, and the enzymatic activity towards the alginate was improved from 53.9 U/mg to 212.86 U/mg compared to Algt. Algt1 was stable when incubated at 40 °C for 90 min, remaining with approximately 80.9% of initial activity. The analyses of thin-layer chromatography (TLC), fast protein liquid chromatography (FPLC), and electrospray ionization mass spectrometry (ESI-MS) demonstrated that the DP of the minimum identifiable substrate of Algt1 was five, and the main hydrolysis products were AOS with DP 1–4. Additionally, 1-L the enzymatic hydrolysis system demonstrated that Algt1 exhibited an effective degradation at alginate concentrations of up to 20%, with the resulting products of monosaccharides (14.02%), disaccharides (21.10%), trisaccharides (37.08%), and tetrasaccharides (27.80%). These superior properties of Algt1 make it possible to efficiently generate functional AOS with low DP in industrial processing.

## 1. Introduction

The cell wall of brown algae contains a significant amount of alginate, which is the most abundant polysaccharide and constitutes 40% of its dry weight [1]. Alginate is found in bacterial biofilms of *Azotobacter* sp. and *Pseudomonas* sp. [2,3,4]. This polysaccharide consists of two isomer residues, β-D-mannuronic acid (M) and α-L-guluronic acid (G), which make up the following three types of building blocks: poly α-L-guluronate (Poly G), poly β-D-mannuronate (Poly M), and Poly MG [5]. In recent years, the degradation products of alginate, known as alginate oligosaccharides (AOS), have been widely studied and applied in multiple fields, including the food industry, agriculture, and pharmacology, owing to their superior bioactivities [6,7,8]. The bioactivity of AOS is affected by its degrees of polymerization (DP) and M/G ratio [8]. Specifically, AOS with DP ≤ 4 show particular pharmacological activities, including anti-obesity [9], antioxidant, anti-apoptosis [10], and the regulation of the lipid metabolism [11]. In addition, AOS rich in M have a stronger immune regulation and antitumor activity [12], while those rich in G exhibit immune regulation and pathogen defense activity [4].

The preparation of AOS by enzymatic hydrolysis of alginate is recognized as an efficient, energy-saving, and environmentally friendly method. The specific activity of alginate lyase plays a crucial role in tuning the efficiency of AOS preparation [10]. Additionally, the production of oligosaccharides at high substrate concentrations results in reduced water and electricity consumption so as to save production costs [13]. Nevertheless, the high viscosity of alginate is considered as a barrier that greatly limits mobility and the substrate-binding ability of alginate lyase [14]. It has been recognized that the common method used to address this problem is to increase the temperature, which is accompanied by a decrease in the substrate viscosity and the improvement of the enzymatic efficiency [15,16]. Therefore, the development of alginate lyases that can efficiently exert enzymatic activity at high-concentration substrate, would be highly advantageous for the industrial production of AOS with low DP.

At present, alginate lyases have been isolated from a wide range of marine and terrestrial bacteria, such as *Pseudomonas* sp. [17], *Flavobacterium* sp. [18], *Sphingomonas* sp. [19], *Pseudoalteromonas* sp. [20], *Microbulbifer* sp. [21], *Alteromonas portus* [22], *Paradendryphiella salina* [23], *Tamlana* sp. [24], and *Vibrio* sp. [25]. The thermostability of the most reported alginate lyases is suboptimal, necessitating the development of alginate lyases with desirable thermostability from the appropriate bacterial sources. The thermophilic bacteria exhibit optimal growth at elevated temperatures and demonstrate greater resilience to extreme environments compared to typical bacteria, thereby rendering their metabolites more stable at high temperatures. It has been proved that enzymes derived from thermophilic bacteria demonstrated superior thermostability, such as *Defluviitalea phaphyphila* [26], *Rhodothermus marinus* [27], and *Vibrio splendidus* [28]. *Microbulbifer thermotolerans* DSM 19189 is an alginate lyase-producing microorganism isolated from the deep sea, which can normally grow in extreme environments [29].

In this study, an alginate lyase from *M. thermotolerans* DSM 19189 was developed and expressed in *Pichia pastoris* GS115, and the non-catalytic domains truncation was adopted to improve the enzymatic activity of the wild-type enzyme. The biochemical properties and hydrolysis products of the recombinant Algt1 were determined to demonstrate its AOS-generating potential. At last, the industrial preparation efficiency of AOS with DP 1–4 was further evaluated at the substrate concentration of 20% at 1-L reaction system.

## 2. Materials and Methods

### 2.1. Strains and Chemicals

*Escherichia coli* TOP 10 and *P. pastoris* GS115 strains were purchased from Sigma-Aldrich (St. Louis, MO, USA) and used for plasmid propagation and expression of enzymes, respectively. Aginate (chemically pure) was purchased from Sinopharm Chemical Reagent Co., Ltd. (Beijing, China). Poly M, Poly G (approximately 95% pure), and standards of various DPs for mannuronate and guluronate were purchased from Bz Oligo Biotech Co., Ltd. (Qingdao, China). All other chemicals and reagents used in this study were purchased from Sigma-Aldrich (St. Louis, MO, USA) and of analytical grade.

### 2.2. Sequence Analysis

To develop a thermostable alginate lyase with elevated enzymatic activity, the non-catalytic truncation strategy was used in this study. The sequences of the full-length original enzyme Algt and the recombinant truncated enzyme Algt1 were analyzed. The open reading frame (ORF) was mapped using the ORF finder (https://www.ncbi.nlm.nih.gov/orffinder/, accessed on 22 May 2022). The signal peptide was predicted using the SignalP 6.0 server (https://services.healthtech.dtu.dk/service.php?SignalP, accessed on 22 May 2022). Conserved domains were analyzed using Conserved Domain Research on NCBI (https://www.ncbi.nlm.nih.gov/Structure/cdd/wrpsb.cgi, accessed on 24 May 2022) and presented using DOG 2.0 [30]. The theoretical molecular weight (Mw) and isoelectric point (pI) of the two enzymes were analyzed by ExPASy (https://web.expasy.org/compute_pi/, accessed on 24 May 2022). A phylogenetic tree of the amino acid sequences of alginate lyases was constructed using MEGA 7.0. Multiple amino acid sequence alignments were accomplished and visualized using Clustal Omega (https://www.ebi.ac.uk/Tools/msa/clustalo/, accessed on 24 May 2022) and ESPript 3.0 program (http://espript.ibcp.fr/ESPript/ESPript/, accessed on 24 May 2022), respectively [31]. The three-dimensional structure of alginate lyase was constructed using the Phyre2 server (http://www.sbg.bio.ic.ac.uk/phyre2/, accessed on 24 May 2022). Molecular docking simulations were performed using Discovery Studio 2.5 software (Accelrys, San Diego, CA, USA) and visualized using PyMOL software (version 1.7.2; Delano Scientific, CA, USA).

### 2.3. Construction of Recombinant Alginate Lyases

The gene of original full-length alginate lyase (Locus_tag: BM155_RS06955) was obtained from the genome of *M. thermotolerans* DSM 19189 (BioProject accession: PRJNA262321), the encoded sequences were deposited in GenBank (accession number WP_231902423.1). The wild-type alginate lyase was named Algt. Additionally, alginate lyase Algt contains three conserved domains, including carbohydrate-binding module (CBM)_4_9, alginate_lyase2, and F5_F8_type_C. Based on the previous work, the truncation of non-catalytic domains, including CBM_4_9 and F5_F8_type_C domains of enzyme markedly enhanced its enzymatic activity, transcription levels, and thermal stability [32]. Therefore, the truncation strategy was adopted in this study. The non-catalytic domains, CBM_4_9 and F5_F8_type_C, of the full-length alginate lyase were truncated, and the truncated alginate lyase named Algt1. The genes encoding Algt and Algt1 were optimized using the software vector NTI 13.5 and were then synthesized and ligated into the vector pPIC9K (pPIC9K-Algt and pPIC9K-Algt1) with the help of General Biosystems Co., Ltd. (Anhui, China).

### 2.4. Heterologous Expression of Alginate Lyases in P. pastoris and Purification

*E. coli* TOP 10 containing the recombinant plasmids was cultured in 20 mL of Luria–Bertani (LB) broth at 37 °C until the absorbance at 600 nm reached 0.8. Plasmids were extracted using the plasmid DNA extraction kit from Omega Bio-tek, Inc. (Norcross, GA, USA). For the expression of Algt and Algt1, the recombinant plasmids pPIC9K-Algt and pPIC9K-Algt1 were linearized and digested using the Sac I restriction enzyme, and the linearized plasmids were transformed into *P. pastoris* GS115 according to the manufacturer’s instructions (Invitrogen) and selected on Minimal Dextrose (MD) agar plates containing 100 µg/mL G418 antibiotic. After incubation at 30 °C for 96 h, colonies were picked from the selected plates and inoculated into 48-pore plates, each containing 1 mL buffered glycerol-complex medium (BMGY) broth. The selected transformants in 48-well plates were cultured at a temperature of 30 °C and an agitation speed of 200 rpm for a duration of 96 h. Methanol was added every 24 h to achieve a final concentration of 1%, thereby inducing the secretion and expression of alginate lyases. The original enzymatic activity of the expressed products was measured, and the transformant with the highest activity was selected for subsequent studies.

The recombinant alginate lyases tagged with 6 × His were purified using Ni^+^-chelated method, according to the manufacturer’s protocol (Suzhou Beaver Biomedical Co., Ltd., Suzhou, China). The concentrations of imidazole in the binding, washing, and elution buffers were notably 5 mM, 20 mM, and 80 mM, respectively. The purified enzymes were further subjected to 12% sodium dodecyl sulfate polyacrylamide gel electrophoresis (SDS-PAGE) for subsequent analysis. Protein concentrations were determined using a BCA protein quantitative analysis kit (Beyotime Institute Biotechnology, Nantong, China).

### 2.5. Enzymatic Activity Assay of Algt and Algt1

The enzymatic activity was quantified by means of using the 3,5-dinitrosalicylic acid (DNS) assay [33]. Briefly, 100 µL of enzyme appropriately diluted was introduced into a solution containing 900 µL of 0.8% (*w*/*v*) alginate (prepared in 20 mM Na_2_HPO_4_-NaH_2_PO_4_ buffer, pH 7.0). The reaction mixture was incubated at 40 °C for 30 min and then terminated by adding 1 mL of DNS solution. Subsequently, the mixture sample was incubated in a boiling water bath for 10 min, followed by the addition of double-distilled water to bring the reaction volume up to a total of 10 mL. Then, the absorbance at 520 nm was measured. One unit of enzyme (U) was defined as the amount of enzyme causing the release of 1 µM of reducing sugar from alginate per minute.

To determine the effect of domain truncation, the enzymatic activity of Algt1 and Algt with same protein concentration was tested after incubation ranging from 30 °C to 55 °C with different pH (4.0–10.0) for 30 min. The highest enzymatic activity of each enzyme was chosen for comparison.

### 2.6. Biochemical Characterization of Algt1

The optimal temperature of Algt1 was determined by assessing the relative enzymatic activity towards alginate, Poly M, and Poly G across a temperature range from 30 °C to 55 °C for a duration of 30 min. To determine the optimum pH of Algt1, the relative enzymatic activity of Algt1 towards alginate, Poly M, and Poly G were tested in CH_3_COOH-CH_3_COONa buffer (20 mM, pH 4.0–6.0), Na_2_HPO_4_-NaH_2_PO_4_ buffer (20 mM, pH 6.0–8.0), and NaOH-glycine buffer (20 mM, pH 8.0–10.0), respectively. The further thermostability of Algt1 was investigated by detecting its residual activity towards alginate after incubation at 35 °C, 40 °C, 45 °C, and 50 °C for 0–2 h. To determine the influence of metal ions and chelators on the activity of enzymes, they were incubated with different metal ions (K^+^, Li^+^, NH_4_^+^, Ca^2+^, Mn^2+^, Mg^2+^, Zn^2+^, Ni^2+^, Cu^2+^, Co^2+^, Ba^2+^, and Fe^3+^) and chelators (SDS and EDTA). Notably, the effect of Na^+^ on them was tested by reacting it with different concentrations of sodium ions (0–1600 mM). Furthermore, the *K_m_* values of recombinant Algt1 towards alginate, Poly M, and Poly G were determined using double-reciprocal Lineweaver–Burk plots based on the initial rates obtained with 0.1–4.0 mg/mL of each substrate in a 20 mM Na_2_HPO_4_-NaH_2_PO_4_ buffer at pH 7.0. To detect the biochemical characteristics of Algt1, the viscosity changes were measured using a viscometer, while the A_235_ changes were measured using a UV detector. The purified and diluted enzyme solution (1 mL, 230 U) was added to 9 mL of 0.8% (*w*/*v*) alginate (20 mM Na_2_HPO_4_-NaH_2_PO_4_ buffer, pH 7.0) and then reacted for 0, 5, 10, 20, 30, 60, 90, or 120 min prior to viscometer and UV detector analysis.

### 2.7. Analysis on Hydrolysis Products of Algt1

According to the results of enzymatic activity and biochemical properties of Algt and Algt1, the hydrolysis products of superior Algt1 were determined to elucidate its degradation pattern. Samples were prepared by incubating 100 μL purified and diluted enzyme solution (10 U) with 900 μL 0.8% (*w*/*v*) alginate (20 mM Na_2_HPO_4_-NaH_2_PO_4_ buffer, pH 7.0) for 1, 3, 10, 30, 60, or 90 min, respectively. The samples were applied onto TLC silica gel 60 F254 sheets (Merck, Darmstadt, Germany) for the thin-layer chromatography (TLC) assay, which were then placed in a solvent mixture containing 1-butanol/formic acid/water (4:6:1, *v*:*v*:*v*). To visualize the hydrolysis product spots, the plate was sprayed with a chromogenic solution. This solution was prepared by dissolving 1 mL of HCl, 2 mL of aniline, 2 g of diphenylamine, and 10 mL of 85% H_3_PO_4_ in 100 mL of acetone. The plate was then heated at 110 °C for 10 min. The hydrolysis products were also detected using fast protein liquid chromatography (FPLC) performed on a Superdex^TM^ 30 Increase 10/300 GL column (GE Healthcare, New York, NY, USA). The mobile phase comprised 0.2 M NH_4_HCO_3_ with a flow rate of 0.3 mL/min and was detected at 235 nm using a variable wavelength detector (VWD). Additionally, electrospray ionization mass spectrometry (ESI-MS) in negative mode was used to illustrate the hydrolysis products of Algt1.

To determine the minimum identifiable substrate of Algt1, using 100 μL mannuronate and guluronate with DP 2–6 as substrates (1 mg/mL), and 100 μL purified and diluted enzyme solution (10 U) was added, then each mixture was incubated at 40 °C for 12 h. The reactants were tested by using TLC assay as previously mentioned. According to the results, the minimum identifiable substrate of Algt1 served as the substrate for docking analysis with Algt1. Docking analyses of Algt1 with alginate pentasaccharide (GGGGG) were performed using the Discovery Studio 2.5 software. Molecular and figure visualization was performed by PyMOL software (version 1.7.2; Delano Scientific, CA, USA).

### 2.8. AOS Batch Preparation at High-Concentration Substrate

To efficiently obtain AOS, 50 mL purified and diluted enzyme solution (5000 U) was added to 1000 mL 20% (*w*/*v*) solutions of different substrates (alginate, Poly M, and Poly G). The reaction mixtures were incubated at 40 °C for 6 h. The reactants were centrifuged at 4000 rpm for 10 min to remove the insoluble substance. Then the collected products were treated by freeze-drying. The recovered hydrolysis products were detected using FPLC, as previously mentioned.

The recovery of the products was determined utilizing the following equation provided:

$$Recovery(\%)=(\frac{W_{1}}{W_{2}})\times 100\%$$

where W_1_ and W_2_ refer to the weight of the degraded products and substrates added initially, respectively.

## 3. Results and Discussion

### 3.1. Sequence Analysis of Algt and Algt1

**Figure 1:**
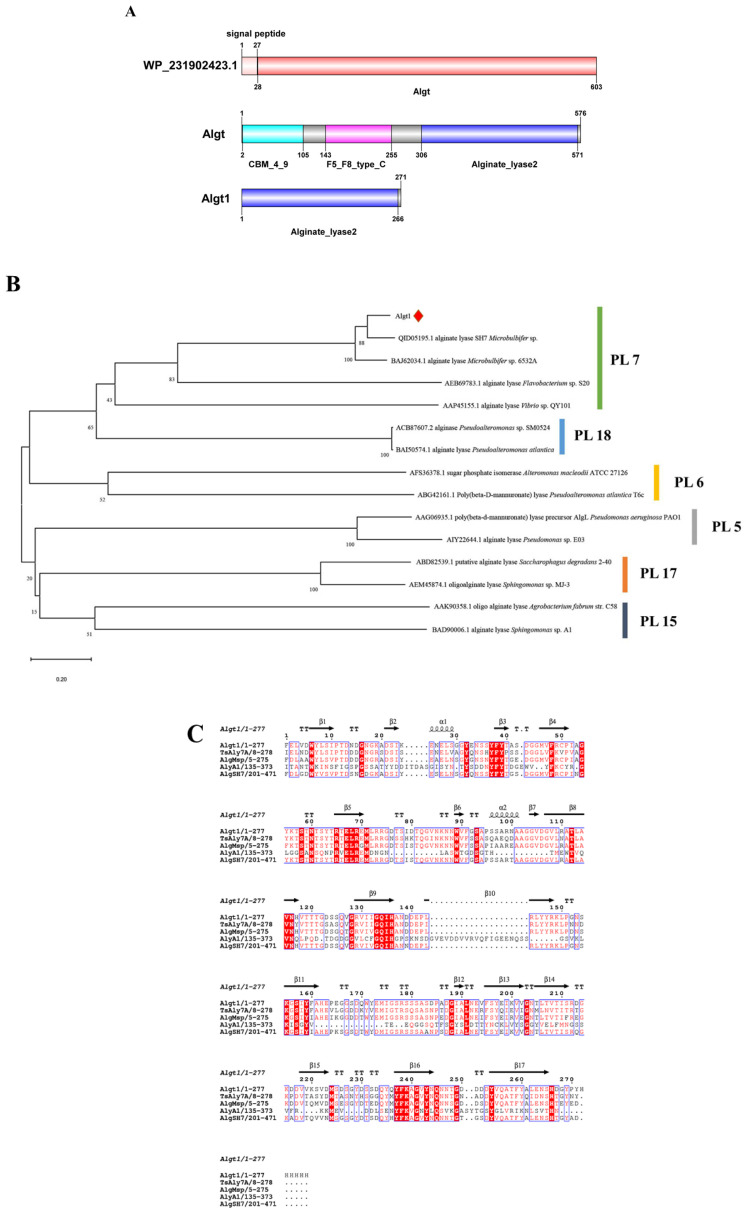
Sequence analysis of Algt and Algt1. (**A**) Conserved domain analysis of Algt and Algt1. (**B**) Phylogenetic analysis of Algt1, bootstrap values of 1000 trials were presented in the branching points. (**C**) Amino acid alignment of Algt1 with other characterized alginate lyases from PL7 family, including TsAly7B (UMF44224) from *Thalassomonas* sp. LD5, AlgMsp (BAJ62034) from *Microbulbifer* sp. 6532A, AlyA1 (URN73184) from *Zobellia galactanivorans*, and AlgSH7 (QID05195) from *Microbulbifer* sp. SH-1. The conserved amino acid residues are highlighted with red backgrounds.

The ORF of the wild-type alginate lyase Algt (WP_231902423.1) from *M. thermotolerans* DSM 19189 was 1812 bp, encoding 603 amino acids. The first 27 amino acids of the complete ORF sequence were identified as signal peptides, and the sequence analysis revealed that the sequence contained no introns. Conserved domain prediction was conducted, and the result was shown in Figure 1A. The original wild-type alginate lyase with no signal peptide sequences was named Algt, and the calculated theoretical Mw and pI values of Algt were 60.75 kDa and 4.24, respectively. The genes of the truncated alginate lyase Algt1 comprised 813 bp and encoded a total of 271 amino acids, devoid of any signal peptide sequences. Furthermore, the calculated theoretical Mw and pI values of Algt1 were 29.66 kDa and 4.50, respectively.

The phylogenetic analysis was employed to establish the relationship between Algt1 and other characterized alginate lyases from PL5, PL6, PL7, PL15, PL17, and PL18 families (Figure 1B). Algt1 forms a robust clade with several documented alginate lyases belonging to the PL7 family, indicating that it represents a novel member of this enzyme family. The amino acid sequence of Algt1 exhibited 85.13% similarity to the well-characterized PL7 family alginate lyase AlgSH7, which was isolated from *Microbulbifer* sp. SH-1 (GenBank: QID05195.1) [23]. The sequence similarity between Algt1 and other characterized alginate lyases belonging to the PL7 family was reflected by multiple sequence alignment analysis. RXEXR, QXH, and YXKXGXYXQ are the common catalytic regions of alginate lyases in the PL7 family (Figure 1C), which play important roles in catalysis. Among the three regions, the key positively charged residues such as Arg (R) and Gln (Q) can neutralize acid groups through the formation of hydrogen bonds with the carboxyl group (COO^−^) of the substrate, and the conserved His (H) and Tyr (Y) residues act as the catalytic base and acid, respectively [21,22]. The phylogenetic analysis and multiple sequence alignment suggest that Algt1 is classified within the PL7 family of alginate lyases.

### 3.2. Heterologous Expression of Algt and Algt1 in P. pastoris and Their Enzymatic Activity

**Figure 2:**
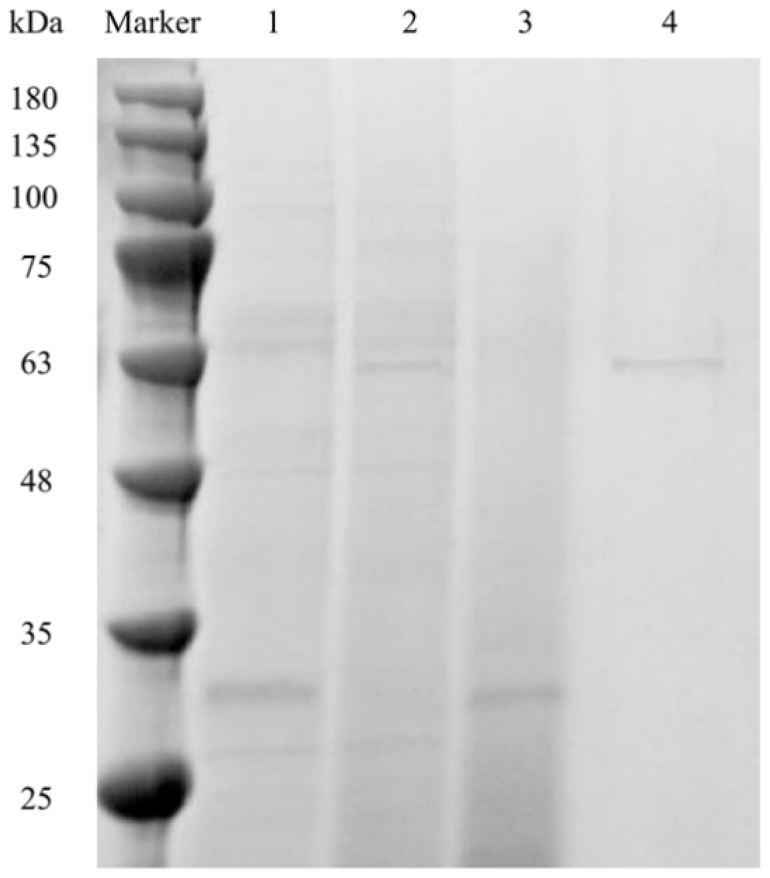
SDS-PAGE analysis of recombinant Algt1 and Algt. Lane M, protein molecular mass marker; Lane 1 and lane 2, crude enzyme solution of Algt1 and Algt from the BMGY medium after 3-d fermentation, respectively; Lane 3, purified recombinant Algt1; Lane 4, purified recombinant Algt.

It was found that truncating the non-catalytic domain of alginate lyase could effectively enhance its enzymatic activity [32,34,35,36]. Therefore, the similar strategy was used to improve the enzymatic activity of the screened heat-stable alginate lyase derived from *M. thermotolerans* DSM 19189. Algt1 and Algt were expressed in *P. pastoris* GS115 with terminal His-tags, then purified. The SDS-PAGE results indicated that Algt1 and Algt were successfully expressed and purified, and the Mw of Algt1 and Algt were approximately 30 and 60 kDa, respectively, consistent with the results obtained from ExPASy (Figure 2).

**Figure 3:**
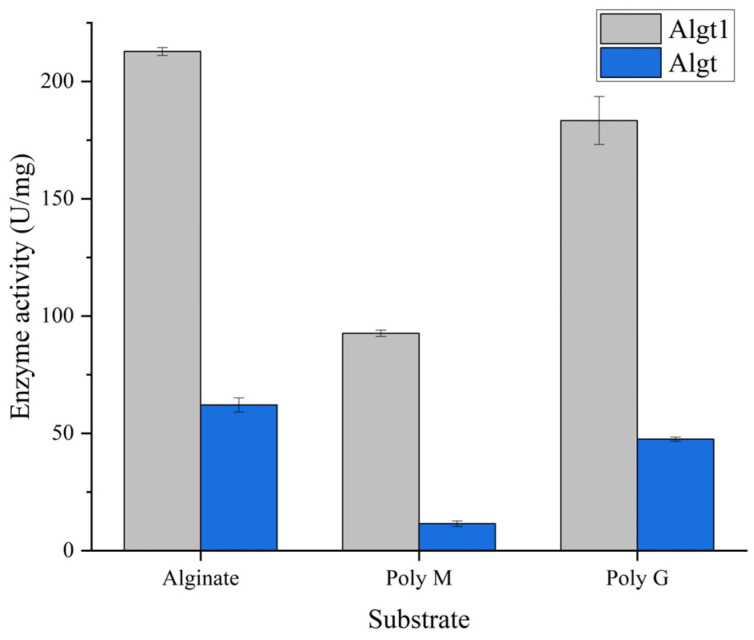
Enzymatic activity of Algt1 and Algt towards alginate, Poly M, and Poly G, respectively.

Through the tests of purified Algt1 and Algt with the same protein concentration, it was found that the enzymatic activity of Algt1 towards alginate, Poly M, and Poly G, were much higher than that of Algt (Figure 3). The enzymatic activity of Algt towards the alginate was 53.9 U/mg, while the truncated enzyme Algt1 exhibited an elevated activity of 212.86 U/mg. Similarly, the enzymatic activity of Algt1 towards poly M and poly G were 8.16 and 3.8 times than the activity of the non-catalytic domains truncated Algt towards the two substrates, respectively. Additionally, Algt1 and Algt exhibit the highest enzymatic activity towards alginate and display a preference for degrading Poly G substrates, indicating that the truncation of the non-catalytic domains does not modify the substrate degradation preference of enzymes. The results demonstrated that the truncation of CBM_4_9 and F5_F8_type_C domains of Algt led to a significant enhancement in its enzymatic activity. Truncating the non-catalytic domains could be considered as a viable strategy for augmenting the enzymatic activity of alginate lyase [32,35,36].

### 3.3. Biochemical Properties of Algt1

**Figure 4:**
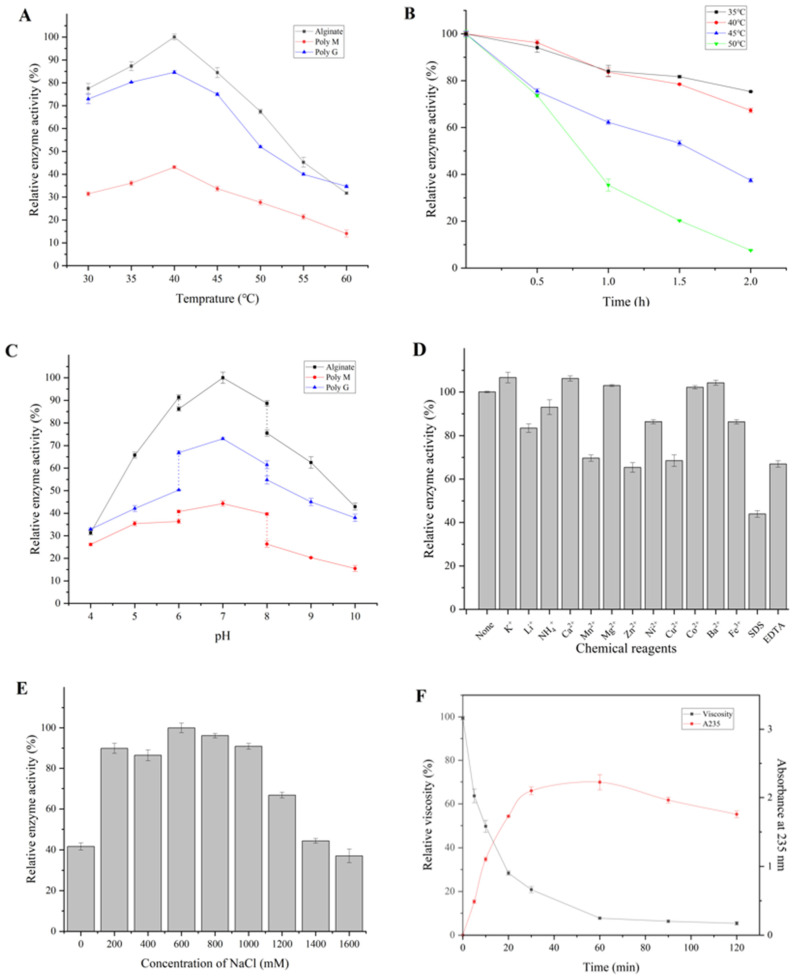
Biochemical characteristics of Algt1. Optimum temperature (**A**) and thermostability (**B**) of Algt1 were determined at different temperatures. Optimum pH of Algt1 was measured in a 20 mM CH_3_COOH-CH_3_COONa buffer (pH 4.0–6.0), 20 mM Na_2_HPO_4_-NaH_2_PO_4_ buffer (pH 6.0–8.0), and 20 mM NaOH-glycine buffer (pH 8.0–10.0), respectively (**C**). Effects of metal ions and chemicals (**D**), and NaCl solution with different concentrations (**E**) on the enzymatic activity of Algt1 were measured. The changes in viscosity and A_235_ values of the reaction solution were monitored (**F**).

The enzymatic properties of the purified Algt1 were further studied. The optimal temperature of Algt1 was 40 °C towards alginate, Poly M, and Poly G. Algt1 still displayed 77.5% and 84.5% of its initial enzyme activities at 30 °C and 45 °C towards alginate (Figure 4A), showing a wide range of adaptive temperatures. Thermostability analysis revealed that the activity of Algt1 was not significantly affected below 40 °C, remaining 80.9% of the initial activities after incubation at 40 °C for 90 min (Figure 4B). As the reaction time increased, the enzymatic activity of Algt1 decreased to 67.3% of the initial activities after heat treatment at 40 °C for 2 h. As an enzyme derived from the thermophilic bacteria *M. thermotolerans* DSM 19189, Algt1 showed a higher optimal temperature range and considerable thermostability compared to the reported alginate lyases (Table 1). For instance, the optimal temperature for AlgL17 was found to be 35 °C; however, it exhibited minimal activity following a heat treatment for 1 h [21]. In addition, the optimal temperature for Alys1 activity was observed at 35 °C, while its capacity to degrade oligosaccharides reduced dramatically exceeded 35 °C [24]. Additionally, VsAly7D exhibited optimal activity at 35 °C; however, this alginate lyase exhibited low activity after incubation above 30 °C [25]. Similarly, Alg17B, which presented high activity at 40–45 °C, exhibited poor thermostability and only remained at 10% activity after treatment at 45 °C for 1 h [37]. The elevated reaction temperature in numerous bioconversion processes can effectively mitigate the risk of contamination, expedite the overall reaction rate, as well as significantly enhance substrate solubility. The relatively considerable thermostability of Algt1 enables it to produce AOS in the industrial environment.

The optimal pH of Algt1 was 7.0, towards alginate, Poly M, and Poly G. Algt1 exhibited a high activity in the pH range of 6.0–8.0, indicating that Algt1 could produce oligosaccharides under neutral pH conditions (Figure 4C). The optimum pH range for some reported alginate lyases is 7.0–8.0, while the reported enzyme PsMan8A has an exceptional optimal pH of 5.0 [30].

The effects of metal ions and chemicals on the activity of Algt1 were measured. Several tested ions, including K^+^, Ca^2+^, Mg^2+^, Co^2+^, and Ba^2+^, slightly increased the enzymatic activity, whereas Li^+^, NH_4_^+^, Ni^2+^, Fe^3+^, Mn^2+^, Zn^2+^, Cu^2+^, SDS, and EDTA made the opposite contribution (Figure 4D). Notably, Ca^2+^ and Mg^2+^ could enhance the activity of some other PL7 family enzymes, such as MtAl138 from *M. thermotolerans* DAU221, which reportedly showed 127% and 105% relative activity in the solution with 1 mM Ca^2+^ and 5 mM Mg^2+^, respectively [39]. Similarly, VxAly7D from *Vibrio xiamenensis* QY104 exhibited 215.14% and 108.27% relative activity in the mixture containing 1 mM CaCl_2_ and MgCl_2_, respectively [40].

In particular, the enzymatic activity of Algt1 was greatly enhanced by the addition of NaCl at different concentrations (200–1200 mM), and the maximum enzymatic activity was observed in the solution containing 600 mM NaCl (Figure 4E). There have been other reports were consistent with the results that the enzymatic activity of alginate lyases could be promoted in the presence of NaCl solution. For example, the enzymatic activity of AlgL17 increased 1.7-fold by the addition of NaCl to a final concentration of 700 mM, while AlgMsp exhibited the highest enzymatic activity at 200 mM NaCl [21,41]. The desirable tolerance to NaCl and metal ions of Algt1 suggested that its enzymatic activity could not be weakening during the production environments.

The enzymatic activity of Algt1 towards alginate, Poly G, and Poly M was 212.86 U/mg, 186.19 U/mg, and 93.87 U/mg, respectively, demonstrating that Algt1 can produce AOS efficiently from various substrates (Table 2). The specific activity of Algt1 exceeded that of numerous previously reported alginate lyases. For example, the specific activity of AlgL17 is 28.99 U/mg, while the specific activity of purified AlgSH17 is 116.8 U/mg [21,38]. The conserved region QIH was detected in Algt1, and the substrate preference of Algt1 aligned with the majority of alginate lyases from the PL7 family, which contained the QIH conserved domain and preferentially degraded Poly G [42]. In contrast, AlgSH7 and AlyPM, containing the QIH domain from the PL7 family, exhibited higher affinity to Poly M than Poly G [43,44]. Therefore, substrate preference cannot be determined only from the difference between the QVH and QIH regions, and the relationship between substrate preference and the presence of specific catalytic domains requires further investigation.

The viscosity of the reaction solution exhibited a significant decrease within the initial 30 min and subsequently maintained only 10% of its initial value at the 120 min mark (Figure 4F), consistent with the classical mode of endo-type alginate lyase [25]. The A_235_ values exhibited a rapidly upward trend during the first 30 min, owing to the formation of conjugate double bonds at the non-reducing end.

### 3.4. Analysis of the Enzymatic Hydrolysates

**Figure 5:**
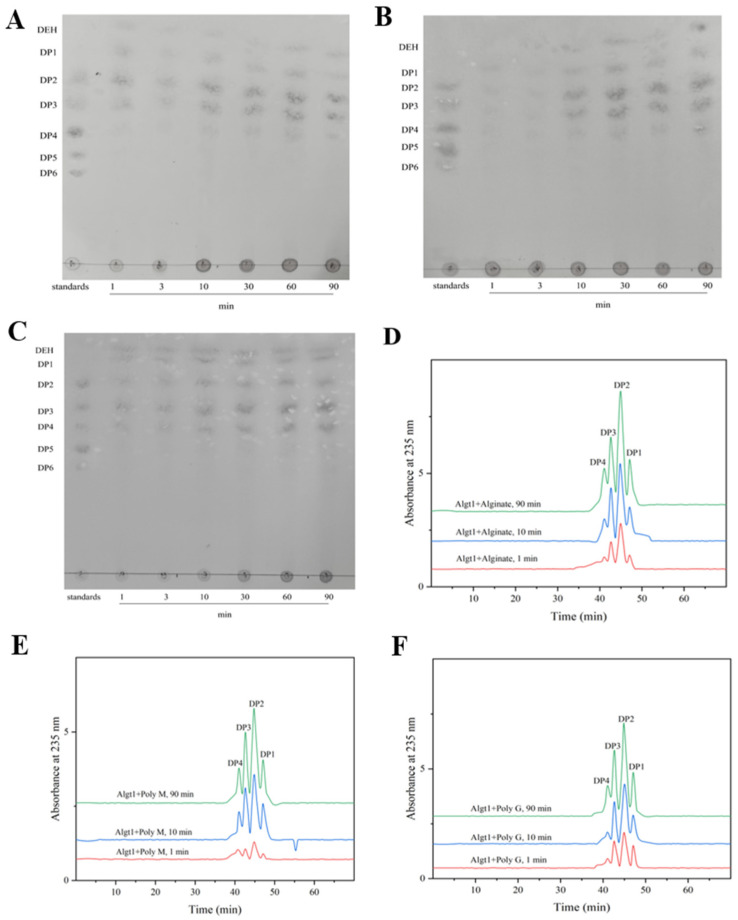
Hydrolysis product analysis of Algt1. TLC analysis of the hydrolysis products of Algt1 with alginate (**A**), Poly M (**B**), and Poly G (**C**). FPLC analysis of action mode of Algt1 towards alginate (**D**), Poly M (**E**), and Poly G (**F**).

The enzymatic hydrolysates of Algt1 were further analyzed. TLC was used to investigate the action pattern of Algt1 under the condition that 100 μL of purified and diluted enzyme liquor with 900 μL of alginate solution reacted at 40 °C. As shown in Figure 5A, the main degradation products of alginate were monosaccharides, disaccharides, trisaccharides, and tetrasaccharides. Additionally, a slight band could be found at the top of TLC, which might be ascribed to 4-deoxy-L-erythro-5-hexoseulose uronic acid (DEH) [40,45]. The enzymatic hydrolysates of Poly M and Poly G were consistent with the results obtained for alginate (Figure 5B,C).

For a better understanding of the hydrolysate products of Algt1, FPLC analysis was further performed. The results of FPLC analyses were presented in Figure 5D, and the main degradation products of alginate were monosaccharides, disaccharides, trisaccharides, and tetrasaccharides. Among them, the disaccharide content was the highest at 52.49%, 43.62%, and 44.21% of the total products after incubation for 1, 30, and 90 min, respectively (calculated by peak areas). Similarly, Poly M could be degraded into monosaccharides, disaccharides, trisaccharides, and tetrasaccharides, and the disaccharides content reached 46.33% of all hydrolysates at 90 min, but few products were detected in the initial. The composition of the Poly G degradation products was similar to that of the degradation products of alginate and Poly M, with the disaccharide content being the highest. During each reaction using the three different substrates, a few oligosaccharides with DP > 4 were detected, indicating that Algt1 efficiently produced AOS with DP 1–4. As for other some reported alginate lyases, the dominant products of AlgL17, Alg17B, and Alg2951 are DP 1–4, DP 1–6, DP 1, and DP 3, respectively [21,22,37]. Although the degradation products of AlgL17 are consistent with those of Algt1, Algt1 exhibits higher enzymatic activity and better thermostability than AlgL17. From the above results, substrates were evidently degraded by Algt1 in both exolytic and endolytic modes, and Algt1 could directionally generate AOS with DP 1–4.

**Figure 6:**
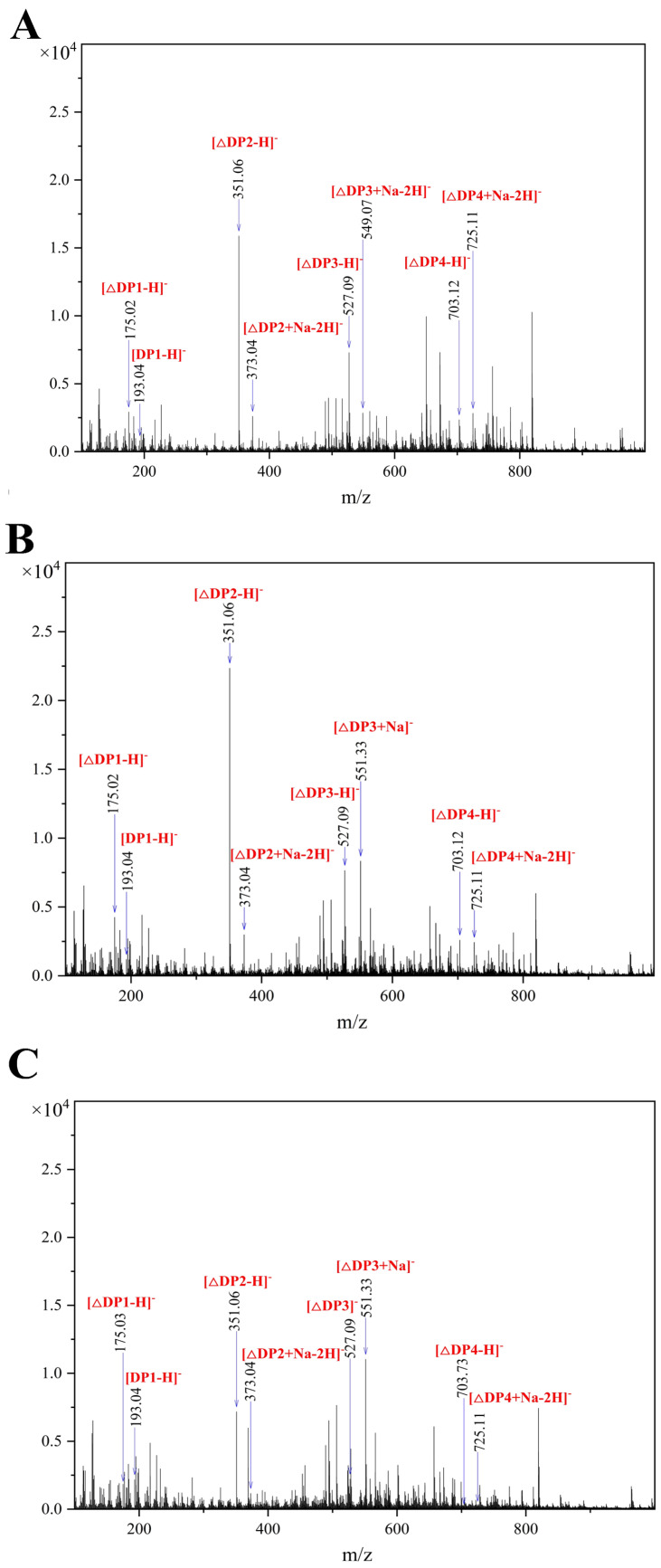
ESI-MS analysis of hydrolysis products with (**A**) alginate, (**B**) Poly M, and (**C**) Poly G, as substrates. DP of the products were distinguished by m/z.

The degrade products of alginate, Poly M, and Poly G by Algt1 were further verified by negative-ion mode ESI-MS analysis. The results showed that the degrade products were monosaccharides, disaccharides, trisaccharides, and tetrasaccharides (Figure 6A–C). Notably, the peak at 175.02 *m*/*z* corresponded to the unsaturated monosaccharide and DEH, while the peak at 193.04 m/z represented a saturated monosaccharide, and the peaks at 351.06 *m*/*z*, 373.04 *m*/*z*, 527.09 *m*/*z*, 551.33 *m*/*z*, 703.12 *m*/*z*, and 725.11 *m*/*z* were related to disaccharides, trisaccharides, and tetrasaccharides, respectively. The ESI-MS analysis also demonstrated the hydrolysis products of Algt1 was the AOS with DP 1–4.

**Figure 7:**
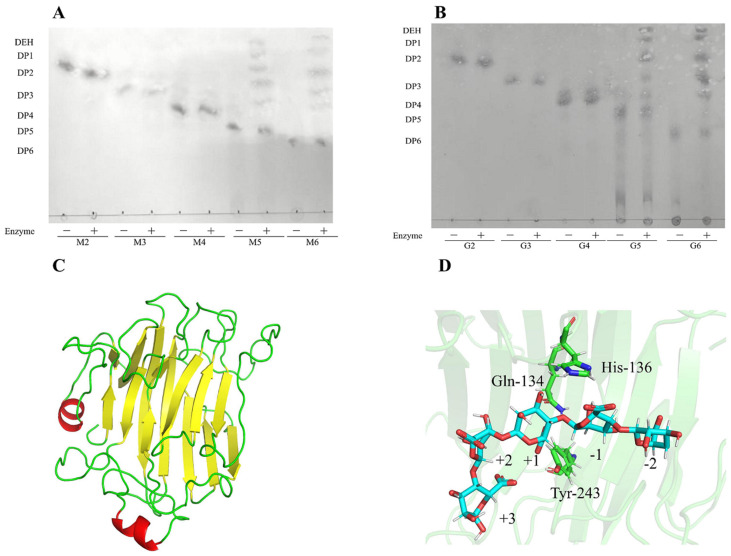
Analysis of minimum identifiable substrate and its molecular docking with Algt1. TLC analysis of the hydrolysis products of Algt1 towards saturated oligosaccharides M2–6 (**A**) and G2–6 (**B**). +, − indicated the addition of enzyme or not, respectively. (**C**) Overall structure of Algt1: β-strands, random coils, and α-rolls are shown in yellow, green, and red, respectively. (**D**) The key residues of Algt1 for binding the minimum identifiable substrate, G5.

TLC assay demonstrated that Algt1 could degrade both D-pentamannuronic acid and L-pentaguluronic acid as the minimum identifiable substrates and converted them to monosaccharides, disaccharides, trisaccharides, and tetrasaccharides (Figure 7A,B). The alginate pentasaccharide was served as the substrate for docking analysis of Algt1.

To comprehensively understand the catalytic mechanism of enzymes, the structure of Algt1 was generated using homology modeling, utilizing the crystal structure of alginate lyase from *K. pneumoniae* (PDB code: 4OZX). The catalytic cavity of Algt1 is composed of β-strands and random coils (Figure 7C). To elucidate the catalytic mechanism of substrate degradation, the combination of Algt1 with alginate pentasaccharide (GGGGG) occupied subsites −3, −2, −1, +1, and +2 were simulated. Based on the results of the molecular docking and His/Tyr β-elimination mechanism of the alginate lyases in the PL7 family [46], Q_134_ possibly attracted COO^−^ in the +1 through a positive charge to ensure proper conformation of the substrate and enzyme (Figure 7D). H_136_ acted as a catalytic base to abstract protons in the +1, while Y_243_ acted as a catalytic base to transfer a proton to the bridging oxygen between –1 and +1. Then, the β, 1-4 glycosidic bond between –1 and +1 broke because of the instability of the structure. This finding provides a valuable reference to the efficient enzyme in the production of AOS.

### 3.5. Potential of Algt1 for AOS Batch Preparation at High-Concentration Substrate

The outstanding thermostability of Algt1 allowed it to continuously degrade substrates at high temperatures, which could increase the fluidity of polysaccharide and facilitate its expansion, thus improving the enzymatic hydrolysis efficiency [47]. In the 1-L batch experiment, 50 mL of Algt1 could efficiently degrade substrate at the concentration of 20% at 40 °C for 6 h. The recovery of degradation products of alginate, Poly M, and Poly G were up to 82.3%, 75.2%, and 79.6%, respectively.

**Figure 8:**
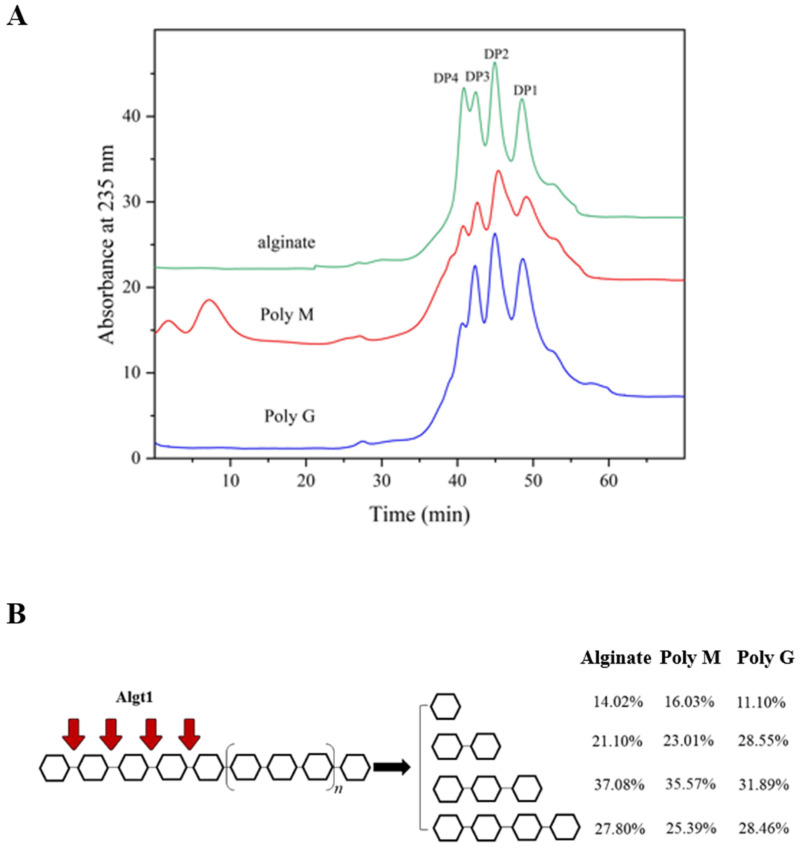
FPLC analysis of final degradation products of Algt1 (**A**) and product distributions pattern (**B**) towards different substrates with the concentration of 20% at 1-L reaction system.

The FPLC analysis was used to detect the end degradation products, and the results showed that Algt1 could degrade alginate, Poly G, and Poly M into monosaccharides, disaccharides, trisaccharides, and tetrasaccharides (Figure 8A). The product distributions pattern was shown in Figure 8B. Algt1 efficiently exerted its enzymatic activity at a high-concentration substrate and generated the AOS with DP 1–4, suggesting its potential for AOS with low DP preparation in the industry. Since AOS with lower DP have been reported to possess pharmacological activities, including anti-obesity [9], antioxidant, anti-apoptosis [10], and regulation of lipid metabolism [11], it can be concluded that Algt1 exhibits potential application prospect.

## 4. Conclusions

A thermostable alginate lyase derived from *M. thermotolerans* DSM 19189 was successfully expressed in *P. pastoris*. The non-catalytic domains truncation strategy proved its feasibility in enhancing the enzymatic activity of the wild-type enzyme Algt. The desirable enzymatic activity and thermostability of recombinant Algt1 enable it to exert the activity and to continually produce the low-DP fragments, even at a substrate concentration as high as 20%. Proved by 1-L enzymatic hydrolysis system, the hydrolysis products of Algt1 are composed of monosaccharides (14.02%), disaccharides (21.10%), trisaccharides (37.08%), and tetrasaccharides (27.80%). It has been recognized that AOS with DP ≤ 4 are bioactive molecules that display antioxidant, anti-apoptotic, and antidiabetic activity, showing wide application prospects. This study first proposed an efficient tool enzyme to produce AOS with DP 1–4 at high substrate concentrations, demonstrating its potential in the industrial preparation of functional oligosaccharides as prebiotics and marine medicines.

## Figures and Tables

**Table 1:** Comparison of the enzymatic properties of Algt1 with some other reported alginate lyases.

Alginate Lyase	Source	Family	Expression System	Specific Activity (U/mg)	Optimal Temperature/pH	Thermostability (Remained Enzymatic Activity)	Substrate Concentration	Hydrolytic Products	Ref.
Algt1	*M. thermotolerans* DSM 19189	PL7	*P. pastoris*	212.86	40 °C/7.0	80.9% (40 °C, 90 min)	20%	DP1–4	This study
AlgL17	*Microbulbifer* sp. ALW1	PL17	*E. coli*	28.99	35 °C/8.0	20% (35 °C, 10 min)	0.5%	DP1–4	[21]
Alg2951	*A. portus* HB161718^T^	PL7	*E. coli*	/	25 °C/8.0	66.5% (40 °C, 30 min)	1%	DP1, DP3	[22]
AlgSH17	*Microbulbifer* sp. SH-1	PL17	*E. coli*	55.62	30 °C/7.0	20% (40 °C, 60 min)	1%	DP1–6	[38]
Alg17B	Strain BP-2	PL17	Native	145	40–45 °C/7.5–8.0	10% (45 °C, 60 min)	2%	DP1–6	[37]
PsMan8A	*P. salina*	PL8	*P. pastoris*	245	25 °C/5.0	ND	0.15%	DP1–2	[23]
Alys1	*Tamlana* sp. s12	PL7	*E. coli*	1350	35 °C/7.0–8.0	70% (50 °C, 60 min)	1%	DP1	[24]
VsAly7D	*Vibrio* sp. QY108	PL7	*E. coli*	663	35 °C/8.0	20% (40 °C, 60 min)	0.3%	DP1–2	[25]

ND means not determined.

**Table 2:** The substrate specificity and enzyme kinetics of Algt1 towards alginate, Poly M, and Poly G.

Parameters	Alginate	Poly M	Poly G
Specific activity (U/mg)	212.86	93.87	186.19
*K_m_* (mM)	0.71	1.38	0.85
*V_max_* (nmol/s)	8.56	5.83	8.09

## Data Availability

The data presented in this study are available from the corresponding author.
